# PASBio: predicate-argument structures for event extraction in molecular biology

**DOI:** 10.1186/1471-2105-5-155

**Published:** 2004-10-19

**Authors:** Tuangthong Wattarujeekrit, Parantu K Shah, Nigel Collier

**Affiliations:** 1 National Institute of Informatics (NII), National Center of Sciences, Hitotsubashi 2-1-2, Chiyoda-ku, Tokyo 101-8430, Japan; 2 Structural and Computational Biology Program, European Molecular Biology Laboratory, Heidelberg, Germany; 3 Max Delbruck Center for Molecular Medicine, Berlin-Buch, Germany

## Abstract

**Background:**

The exploitation of information extraction (IE), a technology aiming to provide instances of structured representations from free-form text, has been rapidly growing within the molecular biology (MB) research community to keep track of the latest results reported in literature. IE systems have traditionally used shallow syntactic patterns for matching facts in sentences but such approaches appear inadequate to achieve high accuracy in MB event extraction due to complex sentence structure. A consensus in the IE community is emerging on the necessity for exploiting deeper knowledge structures such as through the relations between a verb and its arguments shown by predicate-argument structure (PAS). PAS is of interest as structures typically correspond to events of interest and their participating entities. For this to be realized within IE a key knowledge component is the definition of PAS frames. PAS frames for non-technical domains such as newswire are already being constructed in several projects such as PropBank, VerbNet, and FrameNet. Knowledge from PAS should enable more accurate applications in several areas where sentence understanding is required like machine translation and text summarization. In this article, we explore the need to adapt PAS for the MB domain and specify PAS frames to support IE, as well as outlining the major issues that require consideration in their construction.

**Results:**

We introduce **PASBio **by extending a model based on PropBank to the MB domain. The hypothesis we explore is that PAS holds the key for understanding relationships describing the roles of genes and gene products in mediating their biological functions. We chose predicates describing gene expression, molecular interactions and signal transduction events with the aim of covering a number of research areas in MB. Analysis was performed on sentences containing a set of verbal predicates from MEDLINE and full text journals. Results confirm the necessity to analyze PAS specifically for MB domain.

**Conclusions:**

At present **PASBio **contains the analyzed PAS of over 30 verbs, publicly available on the Internet for use in advanced applications. In the future we aim to expand the knowledge base to cover more verbs and the nominal form of each predicate.

## Background

We are now in an era where full genomes, data from high throughput experimental methods (e.g. micro-arrays) and electronic versions of scientific literature are easily available to every researcher over the Internet. These advances have made it possible to work on more than one gene at a time, ask complex questions and increase the pace of biological discovery. However, the progress made in scientific research until now has been recorded in the form of free-text articles readable only by humans and accessible by machine mostly through shallow keyword-based search engines. For improved methods of information access and knowledge discovery it is necessary to automatically map from the unstructured text representation into partially structured forms that provide discovered facts to databases.

The large-scale data generated from the experiments in molecular biology needs to be assessed and integrated into the scientific communities' knowledge stores. This has created a need for various kinds of specialized databases. While some existing databases contain only molecular level information (e.g. PDB [1], SCOP [2]) others (e.g. BIND [3], SWISS-PROT [4], MINT [5]) contain literature associated with molecular entities. These literature databases contain a higher level of relationships (e.g. functional modules, interaction networks, gene products and disease phenotypes), are more informative and can be mined for further knowledge discovery (e.g. G2D [6]). At the same time hand curation of these databases is limiting their growth and reducing the accuracy of the information provided. This is where information extraction (IE) has an important role to play.

Previous research in IE for biology has focused intensively on the recognition of named entities (NE) from scientific texts [7-9], i.e. the identification and classification of technical terms such as proteins, genes, drugs or cell types. Recently, the focus of research has been moving to higher levels of IE such as co-reference resolution and event extraction [10-18] which involves the filling of an event template that makes use of the results from NE recognition. However, significant challenges remain at all levels of biology IE due to the complexity of biological terminology and sentence structure. From the early days of research into computational linguistics it has been known that scientific sublanguages have special properties that make them different from general language [19]. These differences are notable at the level of vocabulary, semantic relationships and sometimes even syntax [20] and often require specialized knowledge sources to aid in analysis. In this article we focus on differences at the semantic and syntactic levels and we will provide motivating examples throughout the following discussion.

Predicate-argument structure (PAS) analysis seeks to formally describe 'frames' for predicates (usually verbs) and the roles of their arguments (parts of the sentence surrounding it). Such roles usually need to be specified according to several factors including meaning and obligation. Meaning can be determined in several ways such as a domain or predicate-specific fashion such as *catalyst *and *reaction being catalyzed *in the case of the first and second arguments to the predicate *catalyze*. Alternatively, functional roles can be employed such as thematic relations that try to express some linguistically motivated aspect of the argument's behavior such as *agent*, *location *or *experiencer*.

Traditional IE systems that use regular expressions based on shallow chunking at the phrase level (e.g. noun phrase, verb phrase, preposition phrase) capture weak notions of 'argument' for event predicates and their linear precedence. Such approaches seem to be inadequate to the goal of achieving high completeness and accuracy in event extraction. In recognition of this several major projects [21-24] have now begun based on newswire and balanced text collections which examine the relations that exist between the constituents in a sentence with the key assumption that those arguments correspond to major objects in events of interest. Although PAS frames seem to be expensive to construct by hand in terms of time and effort, particularly where this requires insights from domain specialists, we believe that this is justified as they provide a systematic reference guide for improving performance compared to ad-hoc pattern-building approaches.

For PAS to be practically realized within IE three major knowledge components will be required: (1) a hierarchy of concept categories for objects of interest; (2) a definition of predicate-argument frames and the semantic labels of their arguments; and (3) the mapping rules that define how to transform the relevant parts of a surface sentence to the arguments in the PAS frame. Currently (1) is already quite advanced with several controlled vocabularies such as MeSH [25] or Gene Ontology [26] now in wide-scale use. At a more modest level core domain specific ontologies for individual annotation schemes such as the GENIA project [27] have also been proposed. To the best of our knowledge, however, nobody has yet made a proposal for (2) which will then serve as the basis on which to develop annotated resources for machine learning approaches to (3). This is the approach we intend to follow and this paper focuses on (2). It is of course possible to approach the task of PAS definition from a machine learning approach, and also to follow a path of hand-built heuristic mapping rules but we believe that both of these approaches may prove to be more costly in terms of time than the one we advocate here.

In this work we introduce the concept of semantic analysis of argument roles in biological texts and propose the construction of PAS for molecular biology (PASBio). We have analyzed and annotated sentences from MEDLINE abstracts and full-text journal articles for building PASBio. The working scheme is similar to the PropBank project [22,23]. Results of our analysis are available online as a knowledge base of predicates and their respective argument sets at PASBio's web page [28]. By specifying PASBio we hope to enhance the event extraction system for accuracy (i.e. the ability to extract only relevant facts) by means of corpus-based semantic interpretation. To achieve this the intended IE system consists of 4 steps: (1) creation of a semantic lexicon (PASBio); (2) semantic annotation of texts using PASBio as a reference resource; (3) building an automatic semantic interpretation model using the annotated texts as a machine learning training corpus; (4) embedding this automatic semantic interpretation module into an IE system. This paper focuses on the key PASBio creation step by discussing the influential processes and choice points and a comparison to other schemes. The annotation task has been done on more than 300 sentences as the result of a preliminary analysis to support in defining PAS frames. This amount of annotation is unlikely to be sufficient for machine learning purposes, so further corpus annotation as well as the machine learning task needs to be carried out in order to reach the final step. It should be noted that other event extraction approaches [14,17,18] and also other text analysis applications (e.g. machine translation (MT), NE recognition tasks, text summarization [29,30]), requiring the use of semantic relations between a verb and its argument in their processing, would be able to take advantages of PASBio.

In this article we first give a short introduction to IE and PAS. Next, we describe the approach taken in the PropBank project. Then, we discuss and exemplify how the specification of predicate-argument frames needs to be extended to meet the requirements for extracting molecular events. The second half of the paper is devoted to explaining the methodology used to define the PAS and discussing results of our analysis and its comparison with those of PropBank. Finally, we describe how the PAS frames can be exploited by showing their place in the IE system for molecular biology and discussing existing IE systems used for event extraction in molecular biology.

## Results and discussion

### Information extraction

IE systems aim to provide instances of structured knowledge representations from unstructured free-form text. IE, based on the Message Understanding Conference (MUC) tradition of task segmentation [31] works fundamentally by using predefined frames and slots in agreement with a specific scenario describing user requirements. Such systems typically use regular expressions to match facts for the event to be extracted in each sentence. Each logical form is founded upon the syntactic relationship between components in each sentence. To take an example from the newswire domain: if we wanted to extract facts relating to a scenario (*company outlook*) then patterns such as "np (stock index) + vp (driven up) + integer (number %)" and "np (company) + vp (bid) + np (stock)" could be developed as a template. Sentences in documents which (1) contain a noun phrase (np) describing *stock index*, together with a verb phrase (vp) *driven up*, and followed by a *number*; or (2) contain a noun phrase representing a *company name*, followed by a verb phrase with *bid*, plus a noun phrase of *stock index *should be extracted. The difficulties are compounded because a single event can nearly always be written in a variety of syntactic forms due to linguistic processes such as passive voice, (pro-) nominalization, raising, etc.

The following simple example involves a linguistic phenomenon sometimes called locative alternation or *spray alternation *by Levin [32]. The verb *spray *may express its arguments in at least two different ways, i.e. (a) "*Peter sprayed water on his flowers.*" and (b) "*Peter sprayed his flowers with water.*" Thus, two syntax-based regular expressions plus some information about NE as "np (people) + vp (spray) + np (object1) + pp (on) + np (object2)" and "np (people) + vp (spray) + np (object2) + pp (on) + np (object1)" are required.

Surface level extraction patterns can be hand built [33] or based on machine learning (ML) from a sample of annotated text (a corpus) [10] or from a few patterns which are known to be good indicators of the topic of interest (seed patterns) [34,35] to reduce the cost and time in constructing patterns manually. However, to extract the relations between objects in the complex sentences that frequently occur in technical and scientific texts requires deeper semantic knowledge. Reported systems [15-18] generally use a set of rules relevant to syntactic roles (e.g. subject, object, and modifier) obtained from parsers together with surface level patterns to extract the interactions between genes or gene products from the biological literature. Although extending the systems with syntactic roles or syntactic functions can achieve better performance compared to the pure pattern-matching approach, some errors resulting from a lack of semantic understanding still remain. For example, [15] mentions that their system will incorrectly extract a protein interaction between "*Msp1p*" and "*Dec1p*" from a sentence "*These findings suggest that Msp1p is a component of the secretary vesicle docking complex whose function is closely associated with that of Dec1p.*", because it conforms to the pattern "A associate with B" predefined within the system. In this respect we consider that deeper knowledge, describing the semantic relationship between verbs and their arguments, encoded in PAS are needed.

### Predicate-argument structures

An event is described in a sentence by a composition of a verb and its arguments. A verb, which indicates a particular type of event conveyed by a sentence, can exist in its verbal form, its participial modifier format or its nominal form. For example, the normal form of a verb used to describe the event "making something active" would be *activate*, its participial modifier format would be *activating *or *activated*, and its nominal format would be *activation*. Beyond a verb, sentence constituents holding semantic roles to complete the meaning of an event indicated by the verb are called arguments. The semantic roles played by the set of arguments with respect to the particular verb are represented in the PAS frame of that verb.

**Figure 1:**
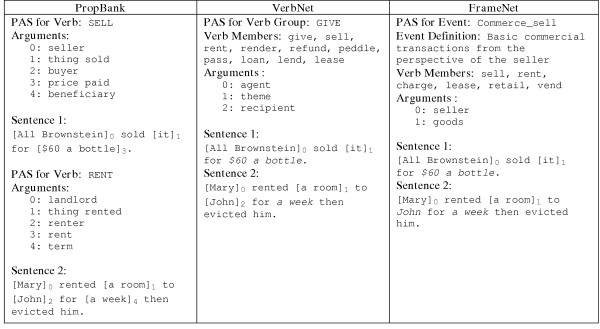
**Predicate-argument structures of PropBank, VerbNet and FrameNet. **The scheme to assign predicate-argument structures can be varied among different projects due to their different focused applications. This figure shows the differences of predicate-argument structures defined from these three projects: PropBank [22, 23], VerbNet [24] and FrameNet [21]. Similar scheme as PropBank is applied to our PASBio project. Discussion about the reason why we are interested in PropBank scheme is discussed in the main text.

Recently several major projects have been proposed that provide resources in the form of an English predicate-argument lexicon. These projects include VerbNet [24], FrameNet [21], and PropBank [22,23]. There are significant differences in approach among these 3 projects. For example, PAS of verbs *sell *and *rent *are proposed as two distinct structures in the case of PropBank and only a single structure for both verbs in the case of VerbNet and FrameNet (Figure 1). VerbNet defines general PAS for a group of verbs that share similar syntactic behavior, underlying Levin's alternations theory [32]. VerbNet's PAS for *give *contains *sell *and *rent *as members. Argument roles for all of the *give *verb members are assigned for *agent*, *theme*, and *recipient *illustrated by example sentences 1 and 2. In the case of FrameNet, PAS is defined based on the underlying principal of what users or applications expect to see for a specific event definition. FrameNet's PAS for event *Commerce_sell *shown in Figure 1 expects only argument *seller *and *goods *from the event driven by any verb in a set of verb members. Considering the annotation on sentence 1 in these 3 projects, "All Brownstein" is annotated as *seller*, *agent*, and *seller *in PropBank, VerbNet, and FrameNet respectively. Similarly, there is also an argument to support the annotation of "it" in all projects. But, only the PropBank scheme has an argument labeled *price paid *to support element "$60 a bottle" of sentence 1 which is likely to be an important participant of the event describing a selling activity. Moreover, a constituent "a week" in sentence 2 is considered to be an argument labeled as *term *only by the PropBank scheme. We consider that arguments like *price paid *for the events involving the verb *sell*, and an argument *term *for events involving the verb *rent*, are important for down-stream user applications. In contrast to VerbNet and FrameNet, PropBank defines individual verb-specific PAS frames which are likely to contain more detailed specifications of arguments than are possible for verb groupings. Moreover, PAS construction in a more verb-specific manner than either VerbNet or FrameNet would assist explicitly in discovering rules for mapping from surface syntactic structures to underlying semantic propositions.

Hence, we utilize PropBank's scheme as a basic starting point and examined sentences containing interesting verbs from a variety of molecular biology journal articles such as MEDLINE abstract [36] and full-text journal articles as EMBO [37], PNAS [38], NAR [39] and JV [40]. The verbs were analyzed and compared to frames proposed by PropBank, which were created based on an analysis of the Wall Street Journal corpus. At least one PAS frame per verb was defined. The verbs were chosen based on both their frequency in the articles and also based on their importance in a number of major event types such as gene expression, molecular interactions and signal transduction.

**Figure 2:**
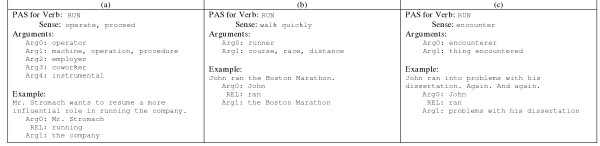
**PropBank's three distinct predicate-argument structures of *run*. **The figure shows examples of predicate-argument structures defined in PropBank [22, 23] project. PropBank defines different predicate-argument structures on account of verb sense variation. Three distinctive predicate-argument structures are defined for the verb *run*. A predicate-argument structure for each sense contains its own set of arguments labeled with semantic roles as shown in the figure.

In PropBank a verb may get more than one PAS frame if the verb sense and its argument set differ, reflecting the fundamental assumption that syntactic frames are directly related to the underlying semantics. For example, PropBank defines three distinctive PAS frames (Figure 2) for the verb *run *on account of sense variation. Each structure contains its own set of arguments labeled with semantic roles. A semantic role of an argument represents a semantic relationship between the argument and its related verb. It is possible that in any particular sentence a complete set of semantic roles or a set of arguments for each sense will not all occur together. The example sentence in Figure 2(a) illustrates this point i.e. only *Arg0 *and *Arg1 *occur in this sentence without the occurrence of *Arg2*, *Arg3*, and *Arg4 *though all arguments are defined as core arguments of the PAS. In each PAS, arguments are labeled ranging from *Arg0 *up to *Arg5 *with a mnemonic label indicating its predicate-dependent role.

Besides these core arguments defined in PAS are adjuncts which are traditionally not defined in PAS because they can potentially take multiple values and not required to minimally define the event. PropBank does consider adjuncts when annotating sentences, and provides labels such as ArgM plus tags such as TMP for temporal information, LOC for locative information, PRP for a reason or motivation, etc. Covering the full working details of PropBank is out of the scope of this paper and we refer interested readers to [22,23] for more information. After manually defining PAS, PropBank has annotated the Penn TreeBank II Wall Street Journal corpus, which contains constituency and dependency information from the TreeBank project [41].

### Events in molecular biology

According to the Gene Ontology (GO) [42], the term *biological process *refers to a broad category of biological tasks accomplished via one or more ordered assemblies of molecular entities (gene products). It often involves transformation, in the sense that something goes into a process and something different comes out of it. Examples of biological processes are cell growth and maintenance, signal transduction, metabolism and biosynthesis etc.

**Figure 3:**
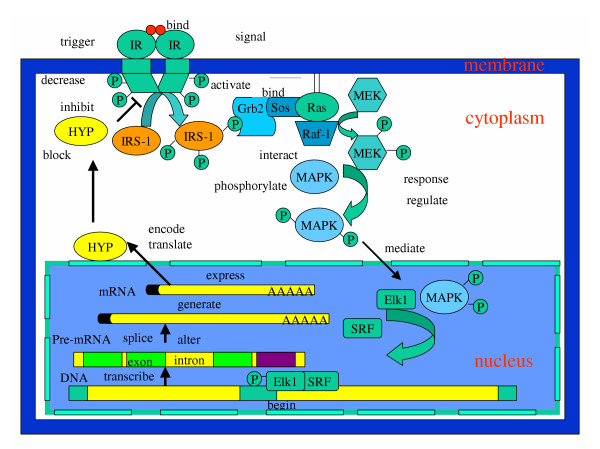
**Molecular events shown by associated predicates. **The figure shows a hypothetical signal transduction pathway of an idealized cell. The signal is triggered at the outer membrane ligand-binding to receptor dimers. This signal is mediated (by various proteins) to the nucleus of the cell using various events (protein-protein interactions, phosphorylation etc.) and initiates transcription of a gene. The protein product (after splicing, translation and synthesis) of the gene inhibits receptor signaling. Thus, it regulates its own expression levels via a negative feedback loop. The direction of information flow is shown with arrows. Cell compartments, molecular entities and predicates describing various events are shown. The predicates analyzed in this work aim to cover events in gene expression, regulation and signaling processes.

A biological process can be subdivided into temporal and spatial molecular events. Each molecular event is carried out by a gene product or well-defined assemblies of them. For example, *phosphorylation *of a protein molecule by a protein kinase is a molecular event, which is a part of the cellular signalling process or *transcription *of a gene by a polymerase is a part of the gene expression process. Hence, by definition a molecular event or a disruption of it will have a local effect in terms of the process that it is a part of and an observable or phenotypic effect in terms of overall effect of disruption of the entire process. For example, a *mutation *in the coding region of a gene that introduces a stop codon into the open reading frame would lead to a pre-mature termination of transcription considered as the local effect and may be responsible for a disease state of an organism due to deficiency of that protein as the phenotypic effect. Different events are described by different verbs (Figure 3) using its associated sets of arguments.

### Need for semantic relationships in molecular event extraction

**Figure 4:**
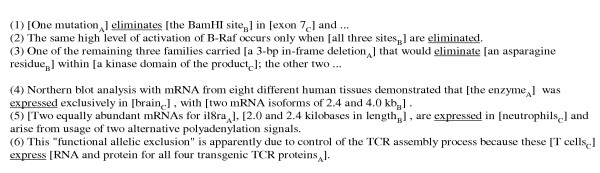
**Example of different forms of *eliminate *and express. **Sentences (1)–(3), three different sentences using predicate *eliminate *taken from MEDLINE [36] and EMBO [37] Journal articles, are given as examples to illustrate the variation of the language usage in biological articles. To convey the information marked as [..._A_] or [..._B_] or [..._C_] can be written in various forms as discussed in the main text. Similarly, the variation of surface linguistic expressions can also be seen from sentences (4)–(6) conveying event *express*. Sentence (6) is an example to show that the domain knowledge is really necessary for correct understanding.

As we exemplified previously for the newswire domain, similar issues of syntactic variants will inevitably be encountered in scientific domains. The following examples from our analysis (Figure 4) illustrate these points.

The sentences (1)–(3) in Figure 4 show some different instances of the event *eliminate *taken from our corpus of MEDLINE [36] and EMBO [37] Journal articles. Here, we consider 3 different pieces of information to be extracted, i.e. A – causal agent of the event, B – the entity being removed, C – location at molecular (sequence) or cellular level where the entity is being removed. In Figure 4, sentence (1) shows simple indicative form of which the syntactic-based extraction pattern would be "A eliminates B in C" (where A = *One mutation*, B = *the BamHI site *and C = *exon7*); sentence (2) shows the passive form, without mention of A and C, for which a syntactic-based extraction pattern would be "B are eliminated" (where B = *all three sites*); sentence (3) shows a form, using a different preposition compared to sentence (1) in order to mention C, for which the syntactic-based extraction pattern would be "A would eliminate B within C" (where A = *a 3-bp in-frame deletion*, B = *an asparagines residue *and C = *a kinase domain of the product*).

Examples of sentences describing the event *express *are shown as sentences (4)–(6). Information slots consist of A – entity expressed, B – physical property of the expressed entity, and C – location referring to organelle, cell or tissue. In sentence (4), (where A = *the enzyme*, B = *two mRNA isoforms of 2.4 and 4.0 kb*, C = *brain*) the information needed to describe the event with respect to slot B is marked by using a prepositional phrase, but in sentence (5), (where A = *two equally abundant mRNAs for il8ra*, B = *2.0 and 2.4 kilobases in length*, C = *neutrophils*) using an appositive form, seemingly not playing an important role in the description of the event in which it participates. Sentence (6), (where A = *RNA and protein for all four transgenic TCR proteins *and C = *T cells*, without mentioning B) shows a different kind of problem that arises because biologists generally would not think of "T cells" as an agent in this context, perceiving it as information about location. On the other hand, without deep domain knowledge one may understand "T cells" as an agent of the express event instead of its intended role as a cell or tissue.

These examples show that using regular expressions around syntactic information of the surface texts would not be adequate for IE to make sense of the complex surface structure. PAS represents information describing verb arguments and the semantic roles these arguments play in conveying a certain event. Different surface forms describing the same event can be mapped into the same PAS.

**Figure 5:**
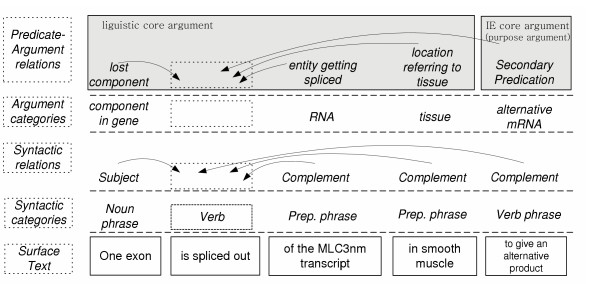
**Syntactic and semantic level description of the surface text. **The understanding makes on the surface text can be shown in different levels. Syntactic categories level gives a syntactical class for each constituent of the sentence. Syntactic relations level describes syntactical function of each constituent of the sentence to predicate of the sentence. Argument categories level offers the semantic concept for each constituent of the sentence. Predicate-argument relation level helps in understanding the semantic role played by each constituent or argument related to its predicate.

To illustrate this point we return to the example mentioned earlier, (a) "*Peter sprayed water on his flowers*." and (b) "*Peter sprayed his flowers with water*." Both sentences can be mapped into the PAS of a verb *spray*, which indicates the particular event "apply thin liquid to surface" with 3 required arguments (i.e. agent, liquid, surface). The sentence's constituent "*Peter*" in both sentences is perceived from its verb-specific semantic role to be an *agent *that does the action. "*water*", when it is either a direct object as in sentence (a) or an object of a preposition as in (b), is perceived as the *liquid *used in the event, and "*his flowers*" is perceived as the *surface *getting wet. Similarly, a surface text from molecular biological corpus such as "*One exon is spliced out of the MLC3 nm transcript in smooth muscle to give an alternative product.*" could be conceptualized into PAS relationship as shown at the topmost level in Figure 5.

Figure 5 illustrates understanding a sentence from the surface text level up to the PAS level. The sentence's constituents "*One exon*", "*is spliced out*", "*of the MLC3 nm transcript*", "*in smooth muscle*", and "*to give alternative product*" have their syntactic categories as *noun phrase*, *verb*, *prepositional phrase*, *prepositional phrase*, and *verb phrase *respectively. At the syntactic relations level, "*One exon*" shows its role as the *surface subject *of the passive form verb "*is spliced out*" and other constituents play the role of *complements*.

Beyond the syntactic level of description, there are semantic levels including argument categories level and predicate-argument relations level. At the argument categories level "*One exon*", "*the MLC3 nm transcript*", "*smooth muscle*" and "*alternative product*" constituents pertain to the domain concept classes of *a gene product (RNA)*, *tissue *and *alternative mRNA *respectively. At the highest level of our scheme the representation contains the most abstract information. Semantic roles played by other constituents to the verb indicating the event are represented at this level. Thus, the process of *removal of an exon from mRNA *(alternative splicing) is indicated by the verb *splice out*. Here, the verb arguments play the semantic roles of *lost component *("One exon"), *entity getting spliced *("the MLC3 nm transcript"), *location referring to tissue *("smooth muscle"), and *secondary predication – showing purpose or reason in this example *("to give an alternative product"). The semantic role *secondary predication *is assigned to the argument "*to give an alternative product*" because this by itself is capable of instantiating a PAS frame and is considered in our scheme to possibly be a core argument.

The semantics of a sentence relate in complex ways to the syntax of the sentence, as we can see from the illustration of semantic and syntactic levels in Figure 5. Using this layered approach different surface forms describing the same event can be mapped into the same PAS. Thus, PAS could be helpful for IE to overcome the syntactic variation problem. After we describe the PAS frames constructed for molecular biology (PASBio), we provide an explanation about how to apply this knowledge in PASBio for event extraction.

### Defining predicate-argument structures for molecular biology

In molecular biology, a gene and its products are at the center of the study, as a set of these molecular entities dictate, and their products carry out, different functions at the cellular level and the combined effects can be seen at the organism level. Hence, in the literature a gene or a gene product is possibly described as an agent participating in some events, with the help of various appropriate verbs indicating the specific events. Different molecular-level or phenotypic effects are described as the other arguments of such events. As described above, PAS is a representation of semantic relationships between arguments with specified roles and a verb relating to a particular event narrated in a sentence. Thus, PAS would be a natural choice for IE, especially event extraction in molecular biology.

#### Guidelines to define PAS

**Figure 6:**
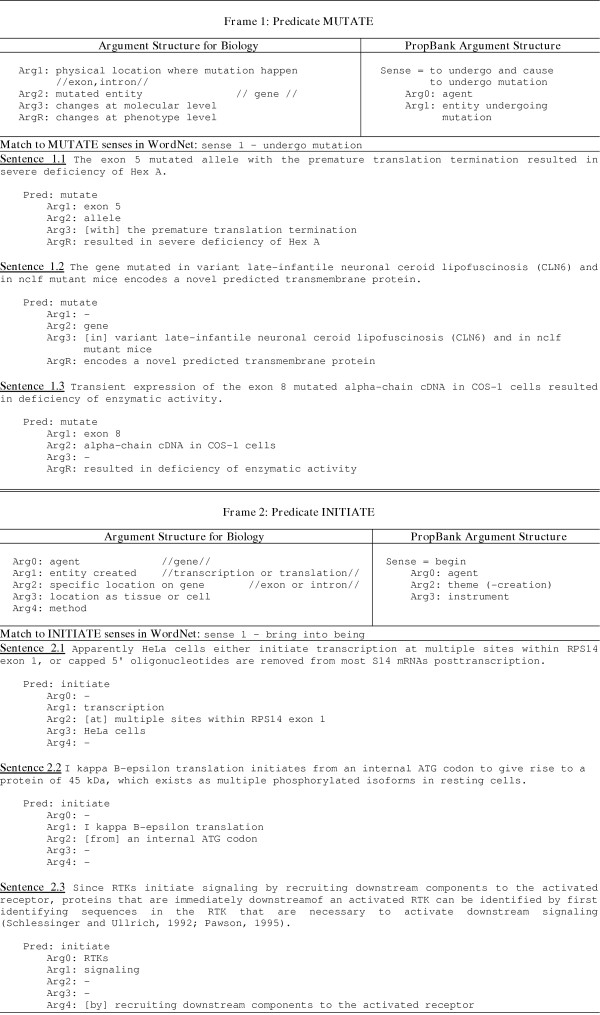
**Examples of predicate-argument structures for group A **As shown by Frame 1, PAS of *mutate *provided in PASBio contains more arguments than as suggested by PropBank [22, 23]. Extra arguments responsible for consequences of the event *mutate *are proposed to be core arguments as they are often seen in sentences from biomedical documents. WordNet [43] sense 1 – *undergo mutation *is correspond to biological sense we found for *mutate*. Three sentences are given to illustrate how surface sentences are mapped into PASBio's predicate-argument structure. Frame 2 shows predicate-argument structure of *initiate *which also belongs to group A – *same sense, more arguments *as same as predicate *mutate*. Extra arguments responsible for spatial information of the event *intitate *are proposed to be core arguments in PASBio, because of their importance from the perspective of biology as discussed in the main text.

We use PropBank's scheme (with necessary adaptations) to define PAS for the molecular biology domain. To define PAS for any verb, a survey about the usages of the verb from a set of sample sentences in a representative corpus is made. Examining the usage of an individual verb will indicate if it needs to be divided into several senses. In PASBio, these senses are divided with the aim of obtaining fine-grained semantic senses using the WordNet [43] lexical database. Each of PASBio's PAS contains a set of core arguments. A core argument is an argument shown by its usage to be important to complete the meaning of the event. Nevertheless, if an argument is considered important but there is no evidence to show that the argument exists together with the predicate in at least 20% of our selected sentences, this predicate may not be assigned as a core argument. There are two different types of core argument: the first type plays a role during the main event denoted by the predicate while the second type plays a role after the main event and aims to express results or consequences of the main event. Further details are given in the next section (Figure 6-Frame 1) illustrated with the PAS for *mutate*. *Arg X *(with *X*, a cardinal number, starting from *0 *and then incremented for each additional argument) is used for labeling the first type of core argument and *ArgR *is used for the second type. A mnemonic label is added after *Arg X *and *ArgR *in order to give a short description of the semantic role played by the argument. Biological function and usage of the argument are used to describe the semantic role in PAS. No attempt is made to ensure the consistency of mapping between argument labels (argument name) and the roles (the mnemonic labels) played by the arguments across verb frames, except *Arg0*. *Arg0 *is reserved for only the argument playing the semantic role of *agent*. In some cases, this agent argument is not found in the usage of some verbs. Thus, PAS frames of such verbs will contain the core argument from *Arg1*. See PAS frames for *mutate *(Figure 6-Frame 1), *express *(Figure 9) and *transform.02 *(Figure 10-Frame 9) as examples.

In addition to annotating a sentence's constituents corresponding to core-arguments with the tag *Arg X *or *ArgR*, the sentence's constituents which do not play the role of core arguments but fall into three types, i.e. adverbial, negation and modality, are annotated with the tag *ADV *or *MAN *in the case of an adverbial, *NEG *in the case of negation, and *MOD *in the case of modality. At the current stage of this project, only adverbials in terms of adverbs are considered to be annotated as *MAN *(for a manner adverb) or *ADV *(for other types of adverbs). If any adverbials in terms of phrases or clauses are mandatory for expressing events indicated by particular predicates, these adverbials will be defined as core arguments within PAS frames. For example, an adverbial phrase playing the role of locative modifier is included in the set of core arguments in the frame for predicate *initiate*. (Refer to example sentence "Apparently HeLa cells either initiate transcription *at multiple sites within RPS14 exon 1*."). Moreover, we are interested in distinguishing only the adverb playing the roles of manner modifiers (e.g. *normally*, *genetically*, etc.) from other adverbs. A manner adverb deserves special distinction from other adverb types because it shows how a certain action is performed which is very important to understand facts in a biological sentence. For example, "*normally*" in the sentence "Mice have previously been shown to develop *normally*" is necessary for IE in order to understand that there is no problem in the development of the mice. Other types of adverbs for example play the roles of aspectual modifiers that give information about whether some event or state of affairs is completed or is still going on, and so forth (e.g. "*still*" in the sentence "Wanda *still *would like to talk about the music festival."), adverbs playing roles as frequency modifiers that indicate the frequency of a certain type of event (e.g. "always" in the sentence "One *always *hears rumors."), adverbs playing roles as focusing modifiers that consist of the four words *even*, *only*, *also*, and *too *(e.g. "The transcription is initiated *only *in female blastoderm embryos."), and so on will be all tagged as *ADV*. In case of negation and modality, *NEG *and *MOD *are given directly to a negator word (i.e. not or n't) and a modal verb (i.e. will, may, can, shall, must, might, should, could and would) respectively. Though negations (operating at the sentence level) and modality (operating at various levels) are not defined as core arguments (mandatory arguments) within any PASBio's PAS frames because linguistically both of them cannot even be considered as any types of predicate's arguments, they are all worth annotating from an IE perspective if they exist in the same clause where a focused predicate exists. Similarly, adverbials which are not mandatory enough to be core arguments are also considered worthy of being annotated when found in the text. We consider that they should not be ignored because they can significantly alter or even reverse the meaning of the sentence.

#### Examples of defined PAS

In this subsection, we show some examples of PASBio's PAS frames and discuss how each frame is defined by examples of sentences relevant to it. There are three important cases that we examine in comparison to PropBank: (1) verbs that are rarely used in general language (e.g. *splice*) or have a unique biological interpretation (e.g. *express*, *translate*, etc.), (2) verbs that have a similar meaning used in the newswire domain and biology domain but show different patterns of usage (e.g. *alter*, *initiate*, etc.), and (3) verbs that are used with the same meaning and usage style in both domains (e.g. *abolish*, *delete*, etc.). The usage of different verbs in biology influence PAS for biological domain falls into four groups: A – same sense, more arguments; B – same sense, fewer arguments; C – same sense, same structure; D – different sense or does not occur. Table 1 shows some verbs for each group. We give PAS of two verbs as examples of each group.

##### Group A

Verbs in this group have been used in biology documents with the same semantic sense as in PropBank, but they required more core arguments in their structures.

Consider the event of mutation, one of the most important biological events and a general cause behind genetic diseases. The verb *mutate *is used to describe the changes in an entity (gene or gene product) and mutations can be natural or engineered. PropBank defines two arguments for this verb which are *Arg0: agent *and *Arg1: entity undergoing mutation*, but from analysis we propose four arguments for the PAS frame of the verb *mutate*. As mentioned in the Guidelines section, *Arg0 *is reserved only for the argument playing the semantic role of agent. From all the examples we observed, passive forms are used to describe *mutate *events which mean that the agent does exist in the event but it is unnecessary to be explicitly stated because it is commonly known by the domain experts. This results in PASBio's core arguments for *mutate *starting from *Arg1 *and we leave a position for agent which possibly could be mentioned in other biological sub-domains. The PASBio's *Arg2 *describing event participating entities (referred to as 'Name Entities') is analogous to PropBank's *Arg1*. Thus PASBio's *Arg1*, *Arg3*, and *ArgR *are extra arguments compared to PropBank. The arguments *Arg1 *and *Arg3 *are captured conforming to linguistic criterion [44] which considers that a sentence element which plays a particular role to a predicate will be considered to be a core argument in a PAS frame even though it does not exist in every sentence in which the predicate appears. In sentences where such an element is omitted we infer that it is implied by the existence of the predicate. For example, in the sentence "John is eating" we infer the existence of a core argument of *eat *which denotes a type of food. Similarly, Figure 6-Frame 1 shows that *Arg1 *and *Arg3 *do not exist in all sentences 1.1 to 1.3, but are assigned as core arguments by their intuitive presence in the domain models of biologists. Noticeably, consequences of the event driven by verb *mutate *are often seen in examples. Apart from "changes at molecular level" assigned as *Arg3*, the consequence, "changes at phenotype level" is suggested as *ArgR *(explained below). Sentence 1.1, 1.2, and 1.3 support this explanation.

The argument *ArgR:results/consequences *is an argument giving information about consequences after the event denoted by the predicate occurs. For *mutate*, most of the example sentences describing this event contain an *ArgR *argument, revealing the necessity of it. The requirement of this argument from an observation perspective coincides with biologist's viewpoint, thus we consider this as a core argument (more precisely an IE core argument) and named as *ArgR *instead of *Arg X *(a core argument from a purely linguistic perspective). We make this distinction under the rule that *Arg X *has to play a role during the event but not after the event. This condition is depicted by a formula like "mutation event = (*Arg X *+ mutation + *Arg X*) + *ArgR*". Empirically, we find that this result argument (*ArgR*) is used with verbs relating to an abnormal biological phenomenon. Examples of other verbs that need this argument are *skip*, *delete*, etc.

Verb *initiate *also takes additional arguments as core arguments. As shown in Figure 6-Frame 2, *Arg2 *(sentences 2.1 and 2.2) describes the point of transcription initiation and *Arg3 *provides information about the tissue/cell where the gene (or product) is expressed. In PropBank, the sentence's segments defined by the parser with functional tag as LOC (location) will be considered as non-required elements. However, the extraction of spatial information is very important from the perspective of biological description. Furthermore, another interesting point that can be seen from the examples in Figure 6-Frame 2 is that authors in biology not only put the agent but also various other kinds of semantic roles in the subject position. In Sentence 2.1 "*HeLa cells*" is syntactically the subject which seems to be the agent of an *initiate *event, but domain knowledge suggests that the agent can be only a protein (usually polymerases bound to the gene being transcribed) in this case. "*HeLa cells*" is annotated as *Arg3:location as tissue or cell *instead of *Arg0:agent*. In sentence 2.2, "*I kappa B-epsilon translation*" is also a subject as in the previous example, but it is "entity created" assigned as *Arg1*. Only in Sentence 2.3 (describing initiation of signaling event), the subject of the sentence fills the semantic role "agent", so a subject "*RTKs*" can be annotated as *Arg0*. Additionally, the point to note is "the entity created" in sentence 2.3 is different from sentence 2.1 and 2.2 as it is a signaling event that is initiated, but not a transcription or translation event.

##### Group B

Verbs in this group have been used in biological texts with the same semantic sense as in PropBank, but they required fewer arguments in their structures in our PAS

**Figure 7:**
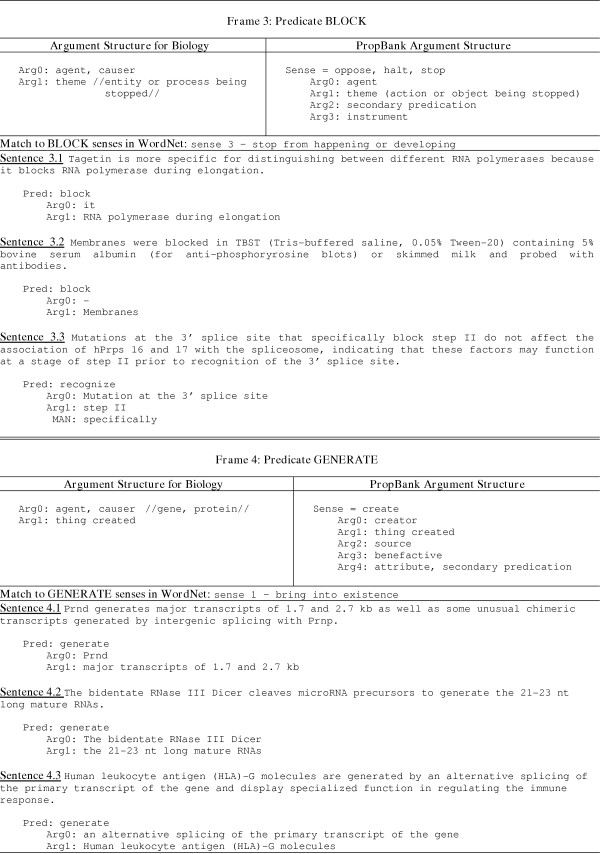
**Examples of predicate-argument structures for group B **Predicate-argument structure for *block*, belonging to group B – *same sense*, *fewer arguments*, is shown as Frame 3. Though *block *is used to mean *stop *in both biological corpus and business news corpus, set of arguments are not the same. Use of *MAN *is illustrated here. Similar to predicate *block*, PASBio's predicate-argument structure of *generate *has less arguments than in PropBank [22, 23] as shown in Frame 4.

Verb *block *both in biomedical texts and in business news texts has very similar semantics. However, an event described by verb *block *in the biomedical domain may not mention information about secondary predication and instrument most of the time. The semantic role *secondary predication *is assigned to the argument that is in itself capable of instantiating another PAS frame. The sentence " [*John*_Arg0_] *blocked *[*Mary*_Arg1_] *from *[*completing her dissertation*_Arg2_] *with *[*his constant pestering*_Arg3_]." is annotated by PropBank's PAS frame. An argument Arg2-secondary predication is annotated for "completing her dissertation" because this contains in itself the PAS of the verb *complete*. From this PropBank example, the meaning of the event denoted by *block *cannot be completely understood if the sentence just states as " [*John*_Arg0_] *blocked *[*Mary*_Arg1_]." as it is necessary to mention the action being stopped. In contrast in the biology domain, by mentioning only the entity being stopped (Sentence 3.1–3.3), the expert reader can understand that the event which applies to that entity is being stopped without providing an explanation of the action being stopped at the position of secondary predication. Similarly, an instrument used to block is encoded in the nature of an agent or causer. The structure of *block *and its examples are given in Figure 7-Frame 3. Only core arguments as defined in the structure exist in Sentence 3.1 and 3.2 (the agent is not mentioned). In sentence 3.3, *MAN *is used to label "specifically" as this adverb plays the role of a manner modifier.

In Figure 7-Frame 4 the PAS frame of *generate *is similar to that of *block*. Only *Arg0-agent *and *Arg1-entity created *are expressed in all observed sentences from our biology corpus.

##### Group C

Verbs in this group have been used in biological documents with the same semantic sense as in PropBank. Moreover, their usage in both the biology corpus and PropBank indicates that their PAS frames are identical. Specialization of domain does not seem to affect verbs in this group.

**Figure 8:**
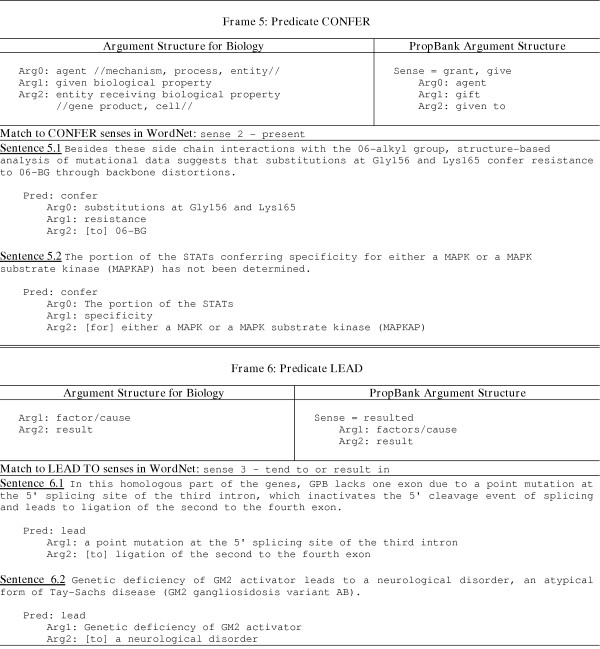
**Examples of predicate-argument structures for group C **Predicate *confer *and *lead *are assigned to group C – *same sense*, *same structure*, so their structures constructed in PASBio are as same as in PropBank [22, 23] as shown in Frame 5 and Frame 6, respectively.

In Figure 8, Frame 5 and Frame 6 show PAS for *confer *and *lead*. In both biology and newswire corpora, *confer *is used with semantic "to give (as a property or characteristic) to someone or something" and *lead to *is used in the sense of "to tend toward or have a result".

##### Group D

**Figure 9:**
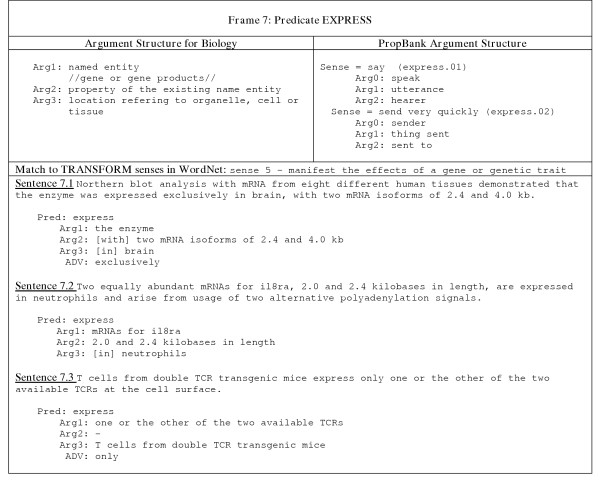
**Predicate-argument structure of *express *(a group D predicate) **Predicate *express *is used in biological documents to mean as WordNet [43] sense 5 – *manifest the effects of a gene or genetic trait *which is totally different from the usage found in business news (i.e. *say *and *send very quickly*). Thus *express *is classified to group D – *different sense or does not occur*.

**Figure 10:**
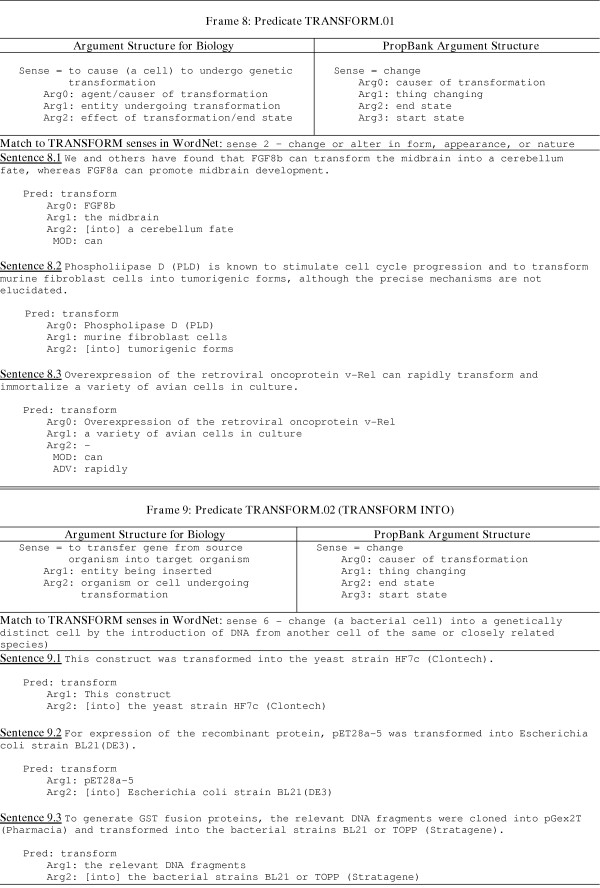
**Predicate-argument structures of *transform *(a group D predicate) **PASBio proposed two different structures for two different senses of *transform *found from the usage in molecular biology corpus. Predicate-argument structure as transform.01 is defined based on the usage with the meaning of WordNet [43] sense 2 – *change or alter in form*, *appearance*, *or nature *and transform.02 is in accordance with the WordNet sense 6 – *change(bacteria cell) into a genetically distinct cell by the introduction of DNA from another cell of the same ore closely related species*.

Verbs in this group have been used in biology documents with a different semantic sense compared to PropBank, or PAS frames for them are not found in PropBank. More than one semantic sense is found in our corpus for some verbs. PAS frames for *express *and *transform *are presented in Figures 9, 10, respectively to illustrate predicate-argument structures for this group.

The verb *express *is used in the biology domain with the meaning "to manifest the existence of a gene or gene product" (or detection of the same by the experimenter) unlike its normal usage with the meaning of "give an opinion or send quickly". The PAS of *express *is given as Figure 9.

In the case of *transform*, two senses are used in biology papers: "to cause (a cell) to undergo genetic (or neoplasmic) transformation" as shown in Figure 10-Frame 8 and "to transfer a gene from source organism into target organism" as shown in Figure 10-Frame 9. Even though the first meaning of *transform *found in our corpus is similar to the sense of "change" found by PropBank, there is still a huge gap between them. In the biological literature, illustrated by examples in sentences 8.1–8.3, this genetic transformation mentions only the agent or causer, what entity is getting transformed, and what will be the effect after transformation. It will not mention the start state of the entity undergoing transformation because it is known from the expert reader's domain 'common sense' knowledge that the start state refers to a normal condition of that entity. *Transform *in the second sense always occurs in a sentence connected by preposition *into*, and in the passive voice form in which no mention is made with regard to the agent.

### Complexities in biology texts

In the discussion so far we have assumed that the predicate is the center of semantic information. Here we intend to show that the argument contents can change the event description specified by the predicate, by examining sentences that describe an 'alternative splicing' event. Alternative splicing is used to generate multiple transcripts from a single gene and hence is a helpful event for increasing the functional complexity of eukaryotic systems.

Consider the following example of a set of sentences that talk about the 'expression' of a single type of mature mRNA generated from 'splicing' of pre-mRNA and generation (and expression) of multiple mature mRNA transcripts with different properties from the single type of pre-mRNA. Sentences annotated follow PASBio's frame for *express*: (a) "*Northern blot analysis with mRNA from eight different human tissues demonstrated that *[*the enzyme*_Arg1_] *was expressed exclusively *[*in brain*_Arg3_], [*with two mRNA isoforms of 2.4 and 4.0 kb*_Arg2_]." and (b) "[*A complementary DNA clone*_Arg1_] *encoding the large subunit of the essential mammalian pre-messenger RNA splicing component 2 snRNP auxiliary factor(U2AF65) has been isolated and expressed *[*in vitro*_Arg3_]." Sentence (a) is considered as a sentence denoting the alternative splicing event but sentence (b) is considered as a negative (not describing alternative splicing) sentence, which talks about expression of an mRNA of a splicing factor.

It would be difficult, based on word contents or regular expression methods, to put these two examples into different 'bins' for alternative splicing events. But the discussion about the length of the two different transcripts in Arg2 (with two mRNA isoforms of 2.4 and 4.0 kb) in the first sentence can be helpful to understand it as a sentence discussing about alternative splicing. On the other hand, the later sentence contains all the interesting words (e.g., mRNA, express and splicing) but misses Arg2, hence describes just an expression event.

### Utilization of PASBio

Construction of PAS frames by expert introspection may be considered as a time-consuming process, however domain-specific PAS frame definitions have valuable uses in several applications as discussed below.

Each PAS frame in PASBio provides a set of semantic relationships between arguments participating in an event and a verb conveying the event. Although we focus on applying PASBio for event extraction in the molecular biology domain, information processing applications that require semantic understanding of a sentence will be able to take advantage of this knowledge. For example, machine translation (MT) requires encoding a surface sentence of a source language into a language independent logical form of clause meaning, and then generating from this logical representation a surface sentence in a target language. PAS would be one practical choice to be used as such a logical representation in MT [29,30]. In the case of a text summarization application, PAS frames could naturally be employed as the basic unit of a discourse representation, before being summarized [45]. PASBio is available online for the wider research community in the molecular biology domain for exploitation in such applications.

With respect to our molecular event extraction system, as we discussed in the introduction, PASBio takes on the role of a reference source in the stage of corpus annotation for creating training examples for machine learning. The planned IE system is composed of 4 activities: (1) construction of PASBio semantic lexicon; (2) annotation of full-text journal in terms of semantic represented in PASBio's frames; (3) construction of the module for automatically transforming an unseen sentence into a logical form of semantic relationships drawn within each particular PASBio frame; (4) integration of the resultant automatic semantic interpretation module within the event extraction system. So far, manual annotation and machine learning have not been completed yet and will be reported elsewhere. For a description of an IE system that can make use of such an annotated corpus we refer readers to the work of for example Surdeanu et al. [46] who uses PAS defined for the newswire domain to extract market change events.

Apart from our corpus-based semantic interpretation approach, several other research groups have proposed systems for event extraction from the biological literature, especially about the interaction information between genes and genes product. Related work so far can be summarized into two sets. The first set of methods use regular expressions and rely on syntactic patterns. These methods may use statistical models of the surface words [12,13], rules of the sentence elements' precedence order [11], shallow knowledge like part of speech tags, syntactic roles of constituents [15,16], gene/protein name dictionaries and domain knowledge (e.g. a template slots for the particular event) about the events they intend to extract [17,18]. A template used in this research group consists of only a simple set of slots for a simple predicate (i.e. the predicate relating only two arguments: subject and object) and only a shallow notion of the predicate-argument structure has been considered (i.e. consider one argument as subject and another as object, but not consider as arguments' semantic roles).

The only work in the second set, that has taken into account a large number of linguistic and deeper semantic aspects is, that of Novichkova et al. [14]. The approach described in Novichkova et al., is to construct a biology IE system (MedScan) containing two components: an NLP engine deducing the semantic structure of a sentence, and a configurable information extraction component to validate and interpret results produced by the NLP engine, in order to achieve a flexible and efficient IE system. In one of its steps, the authors propose to transform the syntactic tree of a whole sentence into a normalized semantic tree, which represents the logical relationships between the words in a sentence. To carry out the transformation, a set of semantic frames describing predicate-argument structures, are required. However, the MedScan system's semantic interpretation process is still under development and not precisely specified.

As mentioned above, most of the approaches, whether a deep notion of predicate-argument relations is taken [14] or a shallow notion [17,18], do require a reference resource of PAS frame for each predicate. In this respect, we believe that PASBio's description of PAS frame for each predicate would make a useful complement to other approaches.

Recently, another research group [47] reported the aim of annotating a biological corpus with semantic knowledge in the form of PAS. While this work appears to be at an early stage it again shows the importance of the definition of predicate-argument frames and the semantics of their arguments as a key knowledge for IE in the molecular biology domain.

## Conclusions

With the explosion of molecular data, tools developed by computer scientists are gradually being applied and integrated in the domain of biology to aid in information access and knowledge discovery. Text data appearing as reports about biological discoveries demands automated IE methods for faster knowledge discovery. Traditional IE systems that use regular expressions based on shallow chunking at the phrase level (e.g. noun phrase, verb phrase, preposition phrase etc.) capture weak notions of 'argument' for event predicates and their linear precedence. Such approaches seem to be inadequate to the goal of achieving high accuracy in event extraction in molecular biology. PAS which is used as a representation of the semantic relationship between a verb and its arguments participating in the event has the potential to support deep knowledge acquisition from a sentence within the extended system framework that is now being proposed within the IE community.

Due to the importance of PAS and the lack of a specific PAS frame resource for the domain of molecular biology, we have proposed the analysis of PAS for molecular biology in this article. We have analyzed sentences for 30 verbs (and different frames per senses of the verb) from MEDLINE abstracts and full-text journal articles where the sentences contain each verb in its verbal form and its participial modified form for building PASBio. Our analysis suggests in some cases a significant difference in the predicate frames compared to those obtained from analyzing news articles by the PropBank project. In addition to the significance of verb senses used in the molecular biology domain, syntactic constructions also differ markedly; such as the use of passives allowing the semantic subject to be omitted where they are part of the common-sense understanding in the domain. Human readers are required to have domain knowledge in order to understand the texts. Our result frames and examples are available to the wider research community as a knowledge base at PASBio's webpage.

In the future, we intend to utilize knowledge from the PASBio frames for annotating a corpus to be used as training examples to achieve automatic annotation of PAS semantics into sentences. Furthermore, we aim to complete analyzing PAS for more verbs related to molecular events and afterwards to extend our analysis to sentences containing the nominal forms of verbs.

## Methods

### Selection of verbs

The English language used in research articles of biological and biomedical sciences is a sublanguage of written natural language. While most of its vocabulary is similar to that of general English, some verbs are domain-specific in nature. Our main focus here is the verbs that are used for describing molecular events in biology. Various researchers have different areas of interest and new concepts are added in the literature continuously. However, the areas of cellular signaling, gene expression, regulation and disruption of expression events are very important for the larger community of investigators involved in basic biomedical research and those involved in high throughput analysis. They are discussed throughout different parts of papers as possible cause of normal and disease states of different organisms. Hence, ignoring the normal distribution (frequency) of different verbs in the literature we choose the verbs from those involved in the above-mentioned processes (events). Most of the verbs are shown in Figure 3.

### Selection of example sentences

Information extraction work is still largely carried out using PubMed abstracts. Using abstracts is advantageous because they contain the highest density of keywords compared to other section of research articles but our intuition is that bio-text mining should scale-up to analyze full journal articles where the most detailed results are contained along with supporting evidence, comparisons to others work and background information, etc. [48] Recent investigations have shown that Introduction and Discussion sections apart from paper abstracts may be viewed as interesting sources of important biological information [49]. We thus define our PAS by analysis on sentences from MEDLINE [36] and from all other sections except the Method section on EMBO [37]. Furthermore, we inspect the usage of some verbs in other journals such as PNAS [38], NAR [39] and JV [40] in order to achieve usage agreement and good PAS. Sentences from the Method section are not used in this analysis as they are limited in terms of biomedical information, have generic written styles and verb sense usage tend to overlap with general language.

Sentences were carefully chosen to cover a broad usage of each verb under study from the MEDLINE and full text journal corpora as described before. We tried to choose equal numbers of sentences containing a particular verb in its verbal format and its participial modifier format. Before starting an analysis on each sentence, a sentence was parsed using Connexor Parser [50] that uses Functional dependency Grammar (FDG), to give parse tree, word, lemma, syntactic function and dependency links between words in order to help in determining the boundary of each argument exists in a sentence. This parse tree served as a useful guide in hand analysis, but was not considered by any means as a gold standard. At least 10 sentences were selected to determine PAS of the verb under study. The use of the parser considerably reduces the manual labors involved in defining arguments.

## Authors' contributions

This work was directed by NC. TW carried out the analysis of the predicate-argument structures with technical support from NC and biological knowledge from PKS. PKS chose the predicates and the sentences analyzed from the MedLine corpus. Sentences from other corpuses were complemented by TW. TW prepared the figures (except fig 3 by PKS). All authors contributed during the whole length of the project and writing of the paper. All authors read and approved the final manuscript.

## Figures and Tables

**Table 1:** Examples of predicates in each group

**Group A : same sense, more arguments**
alter, begin, develop, disrupt, inhibit, initiate, mutate, proliferate, skip
**Group B : same sense, less arguments**
generate, block, decrease, lose, modify
**Group C : same sense, same structure**
abolish, confer, eliminate, lead to, result, delete
**Group D : different sense or not occur**
splice, express, truncate, translate, encode, transform, catalyze, transcribe, recognize

## References

[B1] Berman HM, Westbrook J, Feng Z, Gilliland G, Bhat TN, Weissig H, Shindyalov IN, Bourne PE (2000). The Protein Data Bank. Nucleic Acids Research.

[B2] Lo Conte L, Brenner SE, Hubbard TJP, Chothia C, Murzin A (2002). SCOP database in 2002: refinements accommodate structural genomics. Nucleic Acids Research.

[B3] Bader GD, Donaldson I, Wolting C, Ouellette BF, Pawson T, Hogue CW (2001). BIND-The Biomolecular Interaction Network Database. Nucleic Acids Research.

[B4] Bairoch A, Apweiler R (2000). The SWISS-PROT protein sequence database and its supplement TrEMBL in 2000. Nucleic Acids Research.

[B5] Zanzoni A, Montecchi-Palazzi L, Quondam M, Ausiello G, Helmer-Citterich M, Cesareni G (2002). MINT: a Molecular INTeraction database. FEBS Lett.

[B6] Perez-Iratxeta C, Bork P, Andrade MA (2002). Association of genes to genetically inherited diseases using data mining. Nature Genetics.

[B7] Collier N, Nobata C, Tsujii J (2002). Automatic Acquisition and Classification of Terminology using a Tagged Corpus in the Molecular Biology Domain. Terminology.

[B8] Fukuda K, Tsunoda T, Tamura A, Takagi T (1998). Towards information extraction: Identifying protein names from biological papers. Pac Sym Biocomput.

[B9] Tanabe L, Wilbur WJ (2002). Tagging gene and protein names in biomedical text. Bioinformatics.

[B10] Alphonse E, Aubin Sophie., Bessieres P, Bisson G, Hamon T, Lagarrigue S, Nazarenko A, Manine A, Nedellec C, Vetah M, Poibeau T, Weissenbacher D (2004). Event-based Information Extraction for the biomedical domain: the Caderge project. Joint Workshop on Natural Language Processing in Biomedicine and its applications.

[B11] Blaschke C, Andrade MA, Ouzounis C, Valencia A (1999). Automatic extraction of biological information from scientific text: Protein-protein interactions. Proc Int Conf Intell Syst Mol Bio.

[B12] Donaldson I, Martin J, de Bruijn B, Wolting C, Lay V, Tuekam B, Zhang S, Baskin B, Bader GD, Michalickova K, Pawson T, Hogue CW (2003). PreBIND and Textomy - mining the biomedical literature for protein-protein interactions using a support vector machine.. BMC Bioinformatics.

[B13] Marcotte E, Xenarios I, Eisenberg D (2001). Mining literature for protein-protein interactions. Bioinformatics.

[B14] Novichkova S, Egorov S, Daraselia N (2003). MedScan, a natural language processing engine for MEDLINE abstracts. Bioinformatics.

[B15] Ono T, Hishigaki H, Tanigami A, Takagi T (2001). Automated extraction of information on protein-protein interactions from the biological literature. Bioinformatics.

[B16] Pustejovsky J, Castano J, Zhang J, Kotecki M, Cochran B (2002). Robust Relational Parsing Over Biomedical Literature: Extracting Inhibit Relations. Pacific Symposium on Biocomputing.

[B17] Rindflesch TC, Rajan JV, Hunter L (2000). Extracting Molecular Binding Relationships from Biomedical Text. 6th Conference on Applied Natural Language Processing (ANLP-NAACL'2000).

[B18] Sekimizu T, Park HS, Tsujii J (1998). Identifying the interaction between genes and gene products based on  frequently seen verbs in MEDLINE abstracts. Genome Inform.

[B19] Harris Z (1968). Mathematical Structures of Language. Mathematical Structures of Language.

[B20] Grishman R (2001). Adaptive Information Extraction and Sublanguage Analysis. Workshop on Adaptive Text Extraction and Mining at the 7th International Conference on Artificial Intelligence.

[B21] Baker CF, Fillmore CJ, Lowe JB (1998). The Berkeley FrameNet project. 36th Annual Meeting of the ACL and the 17th International Conference on Computational Linguistics (COLING-ACL 1998).

[B22] Kingsbury P, Palmer M (2002). From Treebank to PropBank. 3rd International Conference on Language Resources and Evaluation (LREC-2002).

[B23] Kingsbury P, Palmer M, Marcus M (2002). Adding Semantic Annotation to the Penn TreeBank. Human Language Technology Conference.

[B24] Kipper K, Dang HT, Palmer M (2000). Class based construction of a verb lexicon. 17th National Conference on Artificial Intelligence (AAAI-2000).

[B25] Nelson SJ, Schopen M, Schulman J, Arluk N (2000). An Interlingual Database of MeSH Translations. 8th International Conference on Medical Librarianship.

[B26] Gene Ontology. http://www.geneontology.org/.

[B27] GENIA Project. http://www-tsujii.is.s.u-tokyo.ac.jp/GENIA/.

[B28] PASBio Project. http://research.nii.ac.jp/~collier/projects/PASBio/.

[B29] Hajic J, Cmejrek M, Dorr B, Ding Y, Eisner J, Gildea D, Koo T, Parton K, Penn G, Redev D, Rambow O (2004). Natural Language Generation in the Context of Machine Translation.

[B30] Han C, Lavoie B, Palmer M, Rambow O, Kittredge R, Korelsky T, Kim N, Kim M (2000). Handling Structural Divergences and Recovering Deropped Arguments in a Korean/English Machine Translation System. Association for Machine Translation in the Americas 2000.

[B31] (1998). DARPA. the Sixth Message Understanding Conference (MUC-7).

[B32] Levin B (1993). English Verb Classes and Alternations: A Preliminary Investigation.

[B33] Hobbs JR, Appelt D, Israel D, Bear J, Kameyama M, Stickel M, Tyson M, Roche E and Schabes Y (1997). Fastus: A cascade finite-state transducer for extracting information from natural-language text. Finite State Devices for Natural Language Processsing.

[B34] Riloff E (1996). Automatically generating extraction patterns from untagged text. 13th National Conference on Artificial Intelligence (AAAI-96).

[B35] Yangarber R (2003). Counter-Training in Discovery of Semantic Patterns. 41st Annual Meeting of the Association for Computational Linguistics.

[B36] MEDLINE Database. http://www.ncbi.nlm.nih.gov/PubMed/.

[B37] The EMBO Journal. http://www.nature.com/emboj/.

[B38] Proceedings of the National Academy of Sciences of the United States of America. http://www.pnas.org/.

[B39] Nucleic Acids Research Articles. http://nar.oupjournals.org/.

[B40] Journal of Virology. http://jvi.asm.org/.

[B41] Marcus M (1994). The Penn Treebank: A revised corpus design for extracting predicate-argument structure. ARPA Human Language Technology Workshop.

[B42] Consortium The Gene Ontology (2000). Gene ontology: Tool for the unification of biology. Nature Genetics.

[B43] Miller GA (1990). WordNet: An on-line lexical database. International Journal of Lexicography.

[B44] Meyers A, Macleod C, Grishman R (1996). Standardization of the Complement Adjunct Distinction. 7th Euralex International Congress.

[B45] Marcu D (2000). The Theory and Practice of Discourse Parsing and Summarization.

[B46] Surdeanu M, Harabagiu S, Williams J, Aarseth P (2003). Using Predicate-Argument Structures for Information Extraction. 41th Annual Meeting of the Association for Computational Linguistics.

[B47] Tateisi Y, Ohta T, Tsujii J (2004). Annotation of Predicate-argument Structure on Molecular Biology Text. Workshop on the 1st International Joint Conference on Natural Language Processing (IJCNLP-04).

[B48] Mizuta Y, Collier N (2004). Zone Indentification in Biology Articles as a Basis for Information Extraction. Joint Workshop on Natural Language Processing in Biomedicine and its Applications.

[B49] Shah PK, Perez-Iratxeta C, Bork P, Andrade MA (2003). Information extraction from full text scientific articles: where are the keywords?. BMC Bioinformatics.

[B50] Tapanainen P, Jarvinen T (1997). A non-projective dependency parser. 5th Conference on Applied Natural Language Processing (ANLP'97).

